# Foxp3+ regulatory T cell therapy for tolerance in autoimmunity and solid organ transplantation

**DOI:** 10.3389/fimmu.2022.1055466

**Published:** 2022-11-17

**Authors:** Jes M. Sanders, Shareni Jeyamogan, James M. Mathew, Joseph R. Leventhal

**Affiliations:** ^1^ Department of Surgery, Comprehensive Transplant Center Feinberg School of Medicine, Northwestern University, Chicago, IL, United States; ^2^ Department of Microbiology-Immunology, Feinberg School of Medicine, Northwestern University, Chicago, IL, United States; ^3^ Simpson Querrey Institute for BioNanotechnology, Feinberg School of Medicine, Northwestern University, Chicago, IL, United States

**Keywords:** solid organ transplantation, allograft tolerance, autoimmunity, regulatory T cells, antigen-specific regulatory T cells, adoptive cell transfer

## Abstract

Regulatory T cells (Tregs) are critical for tolerance in humans. The exact mechanisms by which the loss of peripheral tolerance leads to the development of autoimmunity and the specific role Tregs play in allograft tolerance are not fully understood; however, this population of T cells presents a unique opportunity in the development of targeted therapeutics. In this review, we discuss the potential roles of Foxp3+ Tregs in the development of tolerance in transplantation and autoimmunity, and the available data regarding their use as a treatment modality.

## Introduction

Surgical advances in the field of solid organ transplantation (SOT) have made SOT the gold standard treatment option for individuals with end stage organ disease. Immunosuppressive (IS) agents have further revolutionized the care of transplant patients and have markedly decreased the rates of acute rejection with increased short term graft survival (1). Still, chronic rejection continues to be a significant problem for patients, and recipients are often subjected to a lifetime administration of immunosuppressants (2). Long term administration of IS compromises the overall health and quality of life for patients, exposing them to a higher risk of developing secondary complications such as cancer, infections and cardiovascular diseases (1, 3). Chronic rejection is a primary driver for reduced graft function over time (4), and patients that experience rejection may require supplementary therapeutic interventions such as re-transplantation. For example, over the last 5-10 years, it is estimated that the incidence of chronic rejection in liver transplant recipients is 2-5%, accounting for approximately 15-20% of graft losses and nearly 14% of all liver re-transplantations (5, 6). This places a huge burden not only on the individual patient, but also on the health care system as a whole and the ever-growing waitlist for available organs. Hence, there is an incessant necessity for the development and improvement of the current post-transplant regimen by minimizing or even totally withdrawing IS treatment.

Autoimmunity occurs after a failure of self-tolerance, which in turn allows auto-reactive effector T or B cells to initiate and propagate the inflammatory cascade. The clinical presentation of autoimmunity varies based on the disease process and individual patient characteristics but is a major cause of patient morbidity and significant contributor to rising health care costs. The prevalence of autoimmune disease is estimated to be between 3-5% worldwide (7), and it is a well-established fact that rates of diagnosis are on the rise. The reported mean annual direct costs per patient with systemic lupus erythematosus (SLE) are somewhere between $17,258 and $63,022 regardless of the presence of nephritis (8). Considering all patients with SLE, this equates to $1.4 to $3.2 billion per year (9), and this is but one of over a 100 distinct disease entities. Although there has been tremendous progress in the treatment of these diseases, development of a cost-effective therapeutic that can establish long term remission is necessary.

While the two processes described above ultimately differ in their pathophysiology, they share the fact that a lack of tolerance results in the manifestation of disease. Tolerance begins during lymphocyte maturation in the bone marrow (B cells) and thymus (T cells) (10). Here, autoreactive cells are either signaled for cell death or placed into a state of anergy preventing their activation at peripheral sites (10–12). Regulatory T cells (Tregs) are simultaneously developing in the thymus and are capable of suppressing autoreactive cells that have escaped to the periphery, thereby maintaining self-tolerance (13). Tolerance can also be “induced” once a naive lymphocyte is exposed to a non-self antigen (14). Both centrally (thymic) and peripherally developed Tregs are critical in mitigating unwanted host inflammatory responses. The role of these cells in the context of human disease has gained significant interest, and these cells are now being utilized in the development of treatment strategies. This review provides an overview of the development and function of Foxp3+ Tregs, highlights the protocols currently used to isolate and expand Tregs for clinical use, and summarizes the available knowledge of Tregs and their applications as a cellular therapy as it relates to autoimmunity and solid organ transplantation.

## Classification and function of Foxp3+ regulatory T cells

Regulatory T cells are responsible for the maintenance of tolerance in humans and are further classified based on their origin of differentiation, cell surface markers, and transcriptional expression. Treg development in the thymus occurs *via* a two-step process (15, 16). The first is a result of strong T cell receptor signaling in CD4+ thymocytes that results in the upregulation of cell surface proteins, such as CD25, leading to the development of Treg progenitor cells. The second step is cytokine dependent and increases the expression of Forkhead box p3 (Foxp3) within these Treg progenitors. The final result is a fully mature Treg that can migrate away from the thymus and promote tolerance. Treg production is not confined to the thymus and also occurs in the periphery after exposure to a non self-antigen such as commensal bacteria, food, or viruses (14). These cells are known as pTregs and develop following repeated antigen exposure with sub-optimal co-stimulation, which eventually leads to the transition of Foxp3- cells into Foxp3+ cells (14, 17, 18). Successful conversion to a regulatory phenotype also requires other stimulatory cytokines such as IL-2 and transforming growth factor beta (TGF-β) (17, 19, 20). CD25, also known as the alpha unit of the IL-2 receptor, is expressed on the cell surface of Tregs and is critical to their function and maturation. As such, it has long been used as an identifying marker for this population of T cells. Further, the IL-7 receptor, CD127, has more recently been used to exclude Treg populations as it was shown that downregulation of CD127 was correlated with a highly purified population of Tregs with significant suppressive capabilities (21).

Intracellularly, common to both of these Treg subtypes is the expression of Foxp3, a transcription factor that is important for the immune regulatory functions of Tregs (22). The Foxp3 gene locus contains a Cp-G rich region in the first intron which is important for gene regulation (23). In tTregs, this Cp-G rich region is almost uniformly demethylated and is known as the Treg specific demethylated region (TSDR). Conversely, in non Treg T cells, this area demonstrates complete methylation (24–26). While Foxp3 is known as the most important transcriptional regulator of Tregs, it is not the only one responsible for the transcriptional signature and phenotypic characteristics of Tregs. Foxp3 interacts with a number of transcription factors including forkhead box, nuclear factor of activated T cells and activator protein-1 (27–29), all of which lead to the downstream expression of CD25 and cytotoxic T-lymphocyte antigen 4 (CTLA-4) transcripts (22, 30, 31). Ohkura et al. demonstrated that Cp-G hypomethylation occurs at Foxp3-dependent Treg associated genes *CD25*, *Ctla4* and *Tnfrsf18* as well as the Foxp3 independent Treg genes *Ikzf4* and *Ikzf2* (31). Demethylation of these genes is important for the complete development of the Treg phenotype, and demethylation patterns have been observed to be similar between tTregs and pTregs (31–33). The transfection of Foxp3 in naïve T cells and transcriptional induction of Foxp3 by exogenous TGF-β results in only a partial Treg signature (34, 35), and Foxp3 negative cells continue to exhibit some Treg-like transcriptional signatures (36). Thus, the immunoregulatory functions of Tregs are complex and involve the interplay of multiple genes to achieve a complete, suppressive phenotype and are not reliant only on Foxp3 as was once thought.

**Figure 1:**
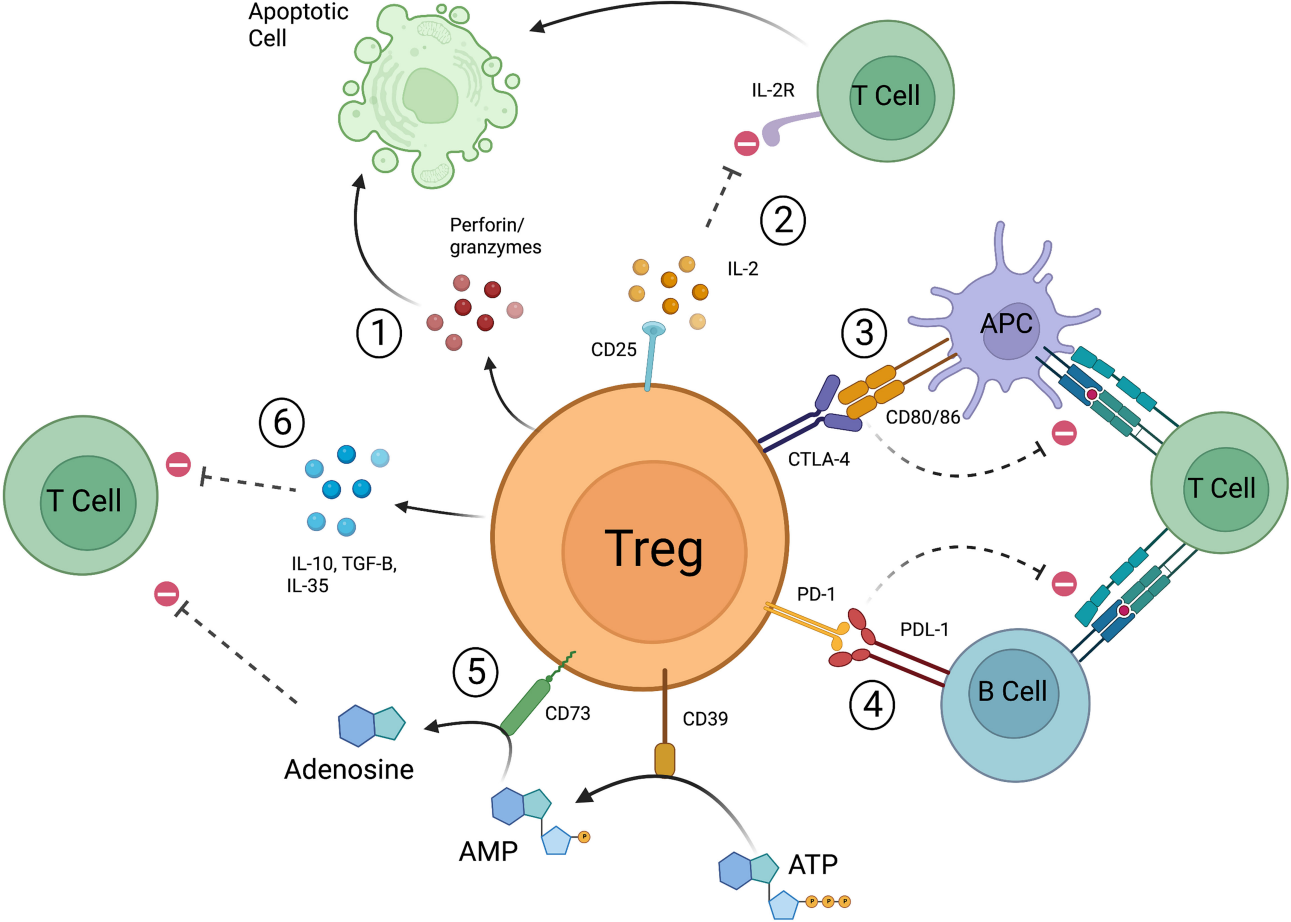
Mechanisms by which Tregs function to suppress pro-inflammatory responses. 1) Release of perforin and granzymes leads to apoptotic pathways. 2) Treg derived CD25 scavenges IL-2, resulting in decreased CD4+/CD8+ effector T cell activation and leads to apoptosis. 3) CTLA-4 expressed on Tregs interacts with co-stimulatory molecules CD80/86 preventing APCs from effectively stimulating effector T cells. CTLA-4 can also directly interact with CD80/86 expressed on effector T cells. 4) Tregs expressing PD-1 prevent autoreactive and alloreactive B-cells from stimulating other T cells. 5) CD39 converts ATP to AMP, CD73 converts AMP to adenosine which then leads to suppression of effector T cells. 6) Tregs release IL-10, IL-35, and TGF-β to promote suppressive action (created with Biorender.com).

Tregs have the ability to not only prevent the transformation of naïve T cells into effector T cells, but to also directly suppress the actions of effector T cells. A number of mechanisms by which this occurs have been described including cytokine deprivation, release of inhibitory cytokines and small molecules, cytolysis, and targeting of antigen presenting cells (APCs) (**Figure 1**). These are not mutually exclusive, and it is likely they all must occur for Tregs to reach their full suppressive potential. Tregs highly express the cell surface marker CD25, which is a subunit of the IL-2 receptor. IL-2 is important for the differentiation and suppressive function of Tregs, but it is also an important factor in the activation of both CD4+ and CD8+ effector T cells (37–40). The much higher affinity of Treg derived CD25 for IL-2 results in IL-2 deprivation mediated apoptosis of effector T cells (39). In this manner, IL-2 and CD25 mediate Treg suppression *via* two pathways—1) through the differentiation of naïve T cell precursors into a mature Treg and 2) through the scavenging of IL-2 resulting in CD4+/CD8+ effector T cell death. Cytolysis is the process by which natural killer cells and cytotoxic T lymphocytes (CTLs) target other cells for destruction through the release of cytotoxic enzymes, granzymes, and perforin (41), but this is not limited to these cell types. Human Tregs also mediate cell death *via* granzyme A and perforin through CD18 adhesion (42), and mouse Tregs have been found to express granzyme B (43, 44). Gondek et al. further studied the reduced suppressive activity of Tregs in a granzyme-b-deficient mouse model (45), and it is now known that Tregs are capable of destroying B cells in a granzyme B and perforin dependent manner (46, 47). Tregs not only secrete factors that result in activation of cell death pathways, but also produce signaling molecules that promote an anti-inflammatory environment. Loss of IL-10 production by Foxp3+ Tregs has been correlated with disease progression in a NOD model of type 1 diabetes (48), and disruption of IL-10 expression in Foxp3+ Tregs led to spontaneous colitis and augmented immune-response related inflammation in the skin and lungs of mice (49). TGF-β produced by Tregs can suppress allergic responses and prevent colitis in inflammatory bowel disease (IBD) models (50, 51), and IL-35, another inhibitory cytokine, is required for maximal Treg suppressive activity (52). Tregs can also increase production of adenosine, an anti-inflammatory molecule, through the actions of CD39 and CD73 (53–55). Finally, Tregs directly interact with APCs to promote tolerance. CD80 and CD86 are known co-stimulatory molecules expressed on the surface of both effector T cells and APCs (56–58). Successful and complete activation of these cell types is reliant not only on recognition of the T cell receptor, but also of these co-stimulatory molecules. CTLA-4 is a cell surface protein that is constitutively expressed on the surface of Tregs and is only expressed on conventional T cells upon their activation. CTLA-4 expressed on the surface of Tregs interacts with CD80/CD86 with higher affinity than CD28 and is able to prevent effective co-stimulation (59). Effector T cells recognizing a specific antigen that do not receive co-stimulation are not fully activated and may be targeted for cell death, and APCs that encounter Treg-based CTLA-4 are unable to promote effector T cell proliferation *via* an increase in tryptophan metabolism (60, 61). Tregs expressing PD-1 have also been shown to interfere with alloreactive and autoreactive B-cells *via* the PDL-1 receptor, again preventing activation of effector T cells (62). Tregs are a unique population of T cells and their ability to promote tolerance is complex and involves a number of inter-related but diverse pathways. As such, much effort has gone into understanding these processes as it relates to human disease and more specifically, autoimmunity and solid organ transplantation.

## The role of Foxp3+ Tregs in autoimmunity and solid organ transplantation

Autoreactive T cells are typically destroyed early during immunological development in the thymus (10). Autoreactive cells that escape to the periphery are kept under control through multiple mechanisms, but the loss of adequate Treg suppression can lead to autoimmunity. Sakaguchi et al. was the first to describe this phenomenon and show that reconstitution of Tregs can prevent the development of such diseases in thymectomized mice (13). The mechanisms by which this occurs though, is still not fully understood. Decreased levels of circulating Tregs have been observed in patients with juvenile idiopathic arthritis (63, 64), hepatitis C virus associated mixed cryoglobulinemia (65), autoimmune liver disease (66), and SLE (67, 68), and lower levels of these cells were correlated with high disease activity and worse prognosis. More recently, fewer Tregs were identified in individuals with rheumatoid arthritis compared to healthy controls and were similarly correlated with higher disease activity (69). However, it must be noted that reduced numbers of Tregs is not uniform across autoimmune diseases, and discrepancies may be due to variability in the markers used to identify Tregs. Given these differences, some researchers have argued that more focus should be paid towards specific sites of inflammation rather than the peripheral blood Treg count. Increased numbers of CD4+CD25+ T cells are known to be recruited to inflammatory sites when compared to peripheral blood (63, 70–72), and these Tregs persist at these sites independent of disease severity and treatment modalities. Leipe et al. advocated for the inhibition of Treg suppressive activity at local sites, and it has been shown that IL-7 and IL-15 were detected in the synovial fluid of patients with juvenile idiopathic arthritis—both of which reduce the activity of Tregs *in vitro* (73, 74). Others have demonstrated that through the inhibition of Tregs at sites of inflammation, responder T cells may continue to promote an inflammatory environment. IL-6 present at inflammatory sites increases the resistance of CD4+CD25- T cells to the suppressive effects of Tregs *in vitro* (75), and it has also been shown to interfere with the migration capacity of Tregs in patients with SLE (76). Yang et al. studied the effects of IL-6 on the Treg and Th17 cell balance in a Th17 reporter mouse model and demonstrated that in the presence of TGF-β, IL-6 in combination with IL-1 induced reprogramming of Foxp3+ Tregs into Th17 effector cells (77). This plasticity has additionally been observed in the setting of active joint inflammation in rheumatoid arthritis (78). Under arthritic conditions, Foxp3+ Tregs transitioned to a Th17 phenotype, a process that was found to be mediated through IL-6 production by synovial fibroblasts (78). However, the role of IL-6 in Treg plasticity seems to be more complex as it has been shown that in the presence of pro-inflammatory cytokines, such as IL-6 and TNF-α, Tregs can be effectively expanded *ex vivo* while maintaining high Foxp3 expression and low secretion of IL-17, IL-4, and IFN-γ (79). IFN-γ can independently affect Treg stability and promote the conversion of Tregs into a Th1-like cell resulting in increased inflammation (80). Alterations in the gene for Foxp3 have been linked to the development of autoimmune disease including IBD and allergic disease in immune dysregulation, polyendocrinopathy, enteropathy and X-linked syndrome (IPEX) humans and scurvy mice (81–84). Deficiency of CD25 correlates with impaired Treg suppressive ability due to decreased IL-10 production which can lead to immunodeficiency and other autoimmune phenomena (85). Clearly, the role Tregs play in the development of autoimmune disease is multi-faceted, and as such creative strategies to utilize these cells in targeted therapeutics must be developed.

**Figure 2:**
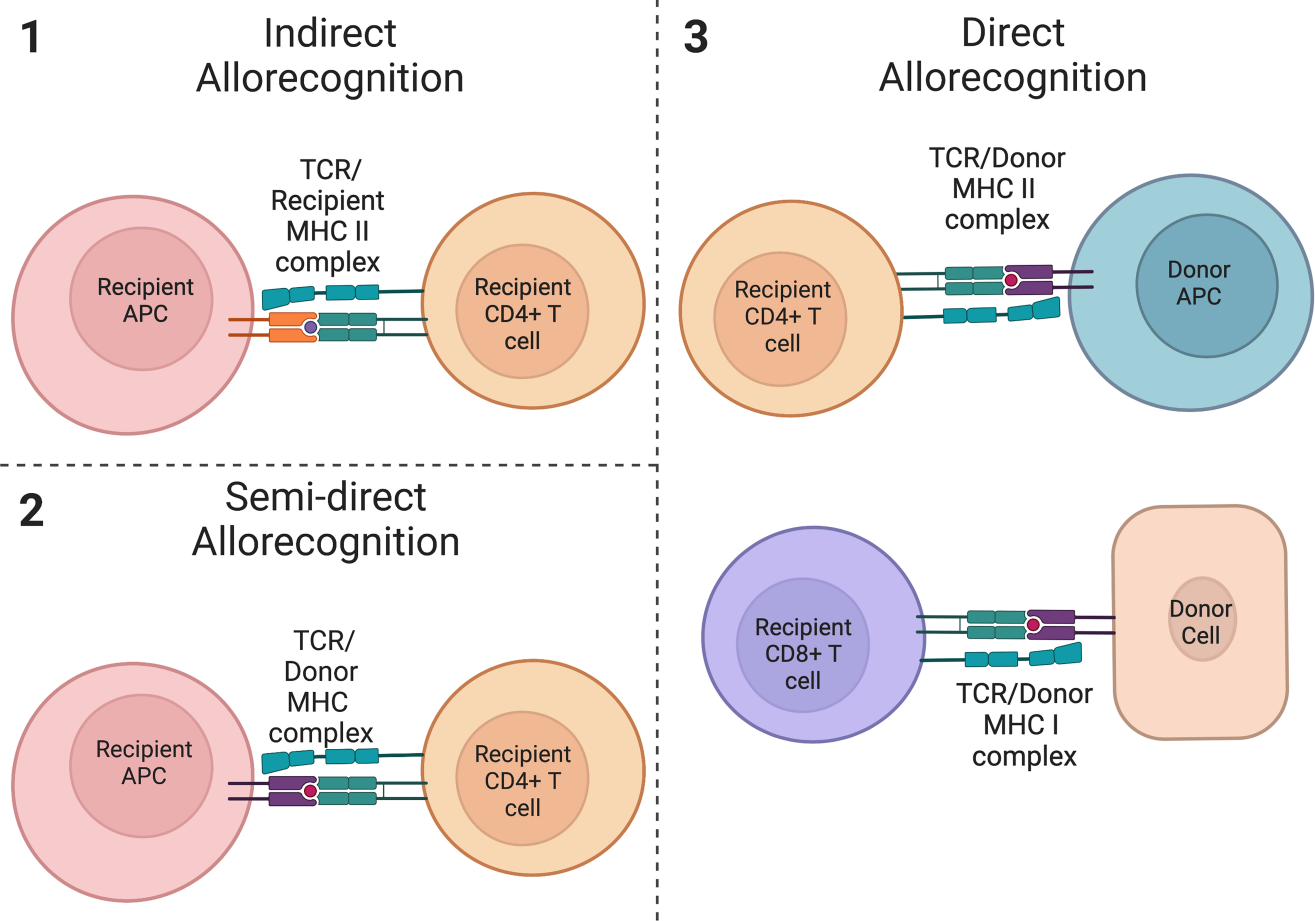
Allorecognition pathways. (1) Indirect allorecognition is activated following presentation of a donor peptide on a recipient APC with a recipient MHC class II molecule. These peptides are recognized by recipient CD4+ T cells. (2) Semi-direct allorecognition occurs after presentation of an intact donor-derived MHC class I peptide by a recipient APC to a recipient T cell. (3) Direct allorecognition occurs following presentation of a donor MHC class II peptide on a donor APC and/or presentation of a donor MHC class I peptide on another donor cell. These peptides are recognized by CD4+ T cells and CD8+ T cells respectively. (created with Biorender.com).

Following a solid organ transplant, the initiation of a series of inflammatory responses leading to the rejection of a transplanted allograft can occur *via* three mechanisms (1): indirect allorecognition (2), semi-direct allorecognition, and (3) direct allorecognition (86–89) (**Figure 2**). The allorecognition cascade is typically triggered after allogeneic molecules are detected by recipient CD4+ T cells (86). Direct allorecognition occurs when donor APCs present donor Major Histocompatibility Complex (MHC) peptides to recipient CD4+ T cells resulting in the destruction of the donor cells (86, 87). On the contrary, the indirect allorecognition pathway is activated following presentation of a donor MHC peptide expressed on the surface of a recipient APC to a recipient CD4+ T cell, again leading to graft injury (86, 87). As it pertains to solid organ transplantation, chronic graft rejection is generally thought to be due to alloreactive CD4+ T cells from the indirect allorecognition pathway whereas acute graft rejection is attributed to alloreactive CD4+ and CD8+ T cells from the direct allorecognition pathway (90, 91). However, the observation of a third pathway challenged conventional thinking and suggests the paradigm of acute versus chronic rejection may be more complex. The semi-direct allorecognition pathway was proposed following the observation that intact MHC:peptide complexes could be transferred across cell types (88, 89, 92). In a mouse skin graft transplant model, Smyth et al. demonstrated the acquisition of donor-derived MHC class I:peptide complexes by recipient dendritic cells up to one month post-transplant which in turn activated CD8+ T cells (89). The group concluded that semi-direct allorecognition is likely a major driver of alloimmunity following organ transplantation. As it relates to graft rejection, the requirement for transfer of donor MHC:peptide complexes to recipient APCs would suggest that semi-direct allorecognition likely contributes to a late response in the rejection continuum. Effector T cells can also activate B cells to produce allo-antibodies which then amplify and propagate the inflammatory process. Thus, a combination of cell and alloantibody mediated immune destruction contribute to graft rejection. Tregs function to suppress the actions of these pathways *via* the mechanisms described above. In humans, Tregs are correlated with allograft survival and have been found to be amplified in the peripheral blood of tolerant allograft recipients (93–98). Foxp3 has also been found to be expressed in rejecting allografts (99) and is associated with fewer chronic changes on biopsy and donor-specific hyporesponsiveness (94), suggesting the importance of Tregs in maintaining graft tolerance and in responding to episodes of rejection.

Although effector T cell recognition of donor antigens can lead to rejection, a unique feature of organ transplantation is that the graft is the most important source of antigen and is also essential in controlling host immune response (100). In a cardiac allograft mouse model, Hamano et al. showed that the allograft was essential in providing donor alloantigens to maintain an unresponsive and suppressive state (100), likely through the actions of Tregs. Other early studies of Tregs showed that the transfer of tTregs and effector T cell progenitors into T cell-deficient mice resulted in the inability of CD4+CD25- cells (non Treg cells) to reject minor and MHC mismatched allogenic skin grafts (101, 102). A number of investigators were able to prevent graft rejection in heart and kidney transplant mouse models in immune naïve mice utilizing polyclonally or alloantigen specific regulatory T cells (103–105). Nadig et al. prevented the development of transplant arteriosclerosis, a hallmark of graft rejection, in humanized mouse models through the actions of *ex-vivo* expanded Tregs (106–108). The use of TCR gene transfer to confer indirect allospecificty on Tregs was studied in partially and fully MHC mismatched, mouse skin and heart allograft transplant models. Infusions of dual specificity Tregs demonstrated longer term survival of allografts and no evidence of myocardial architectural change on histology when compared to Tregs that recognized MHC molecules only through the direct pathway (109). Rejection of renal allografts has been prevented in non-human primate models through the administration of *ex-vivo* expanded antigen specific Tregs (110, 111). Alloantigen specific Tregs have also prevented rejection of skin grafts in humanized skin graft models (112). Alloantigen specific Tregs were shown to more effectively prevent rejection of skin grafts when compared to polyclonally expanded Tregs, with histological analyses of skin grafts demonstrating near normal architecture in mice that received the antigen specific Tregs (112, 113). Tregs are not limited to the CD4 compartment, as Zhou et al. showed that the generation of intragraft CD8+ Tregs was correlated with prolongation of graft survival in a rat kidney transplant model (114). Additionally, both natural killer T cells in a mouse cardiac allograft model, and TCR+CD4-CD8- Tregs in a skin allograft transplant model promoted tolerance and prevented rejection of grafts (115, 116). However, much is still unknown regarding these non-CD4 Treg subsets, and they are not the focus of this review. Given their substantial role in maintaining immune homeostasis, Tregs have been extensively studied and technologies have been created to leverage these cells for clinical use.

## Foxp3+ Treg adoptive cell transfer technologies

Tregs have the potential to revolutionize the care of transplant patients and those with autoimmune disease, but two major hurdles exist in the widespread application of this cellular therapy—1) the ability to reliably expand these cells for adoptive transfer and 2) understanding how *in-vitro* studies correlate with *in-vivo* function. The first evidence for the use of regulatory Tregs in the treatment of human disease was in 2009 (117). In this study, Tregs were isolated using immunomagnetic and fluorescence activated cell sorting (FACS) followed by a 3-week expansion in the presence of IL-2 and anti-CD3/anti-CD28 beads. Modifications of this protocol have been developed, but the basic algorithm has remained largely unchanged over the last two decades. The first step is universal and requires isolation of Tregs from a source. The most feasible and reproducible source of Tregs is peripheral blood. More recently, umbilical cord blood is being used more often as the rates of cord blood banking increase (118). Additional source material for the isolation of Tregs is the thymus from pediatric patients, such as during a heart transplant, given the high density of Tregs in the thymus and lack of CD4+CD25+ effector T cells (119). Other sources of Tregs currently under investigation include induction of pluripotent stem cells (120), induction of conventional T cells with IL-2, rapamycin and TGF-β (121), forced expression of FoxP3 in conventional T cells, and ectopic expression of Foxp3 in an effort to reprogram conventional T cells (122). Once a source is identified, the Tregs must then be isolated. Traditionally, either CD25+ immunomagnetic beads with or without FACS is used for cell sorting and isolation as described in Trzonkowski’s 2009 study. But others have utilized different methods including both CD8+ and CD19+ depletion followed by CD25+ isolation (123–126). Further adjustments have been made given the concern for selection of effector T cells that express both CD4 and CD25, as it has been shown that activated T cells will outgrow Tregs in a prolonged culture (127). Downregulation of CD127 is now being used as a marker for Tregs when utilizing FACS (117, 128, 129) in an effort to create as pure a population of Tregs as possible.

**Figure 3:**
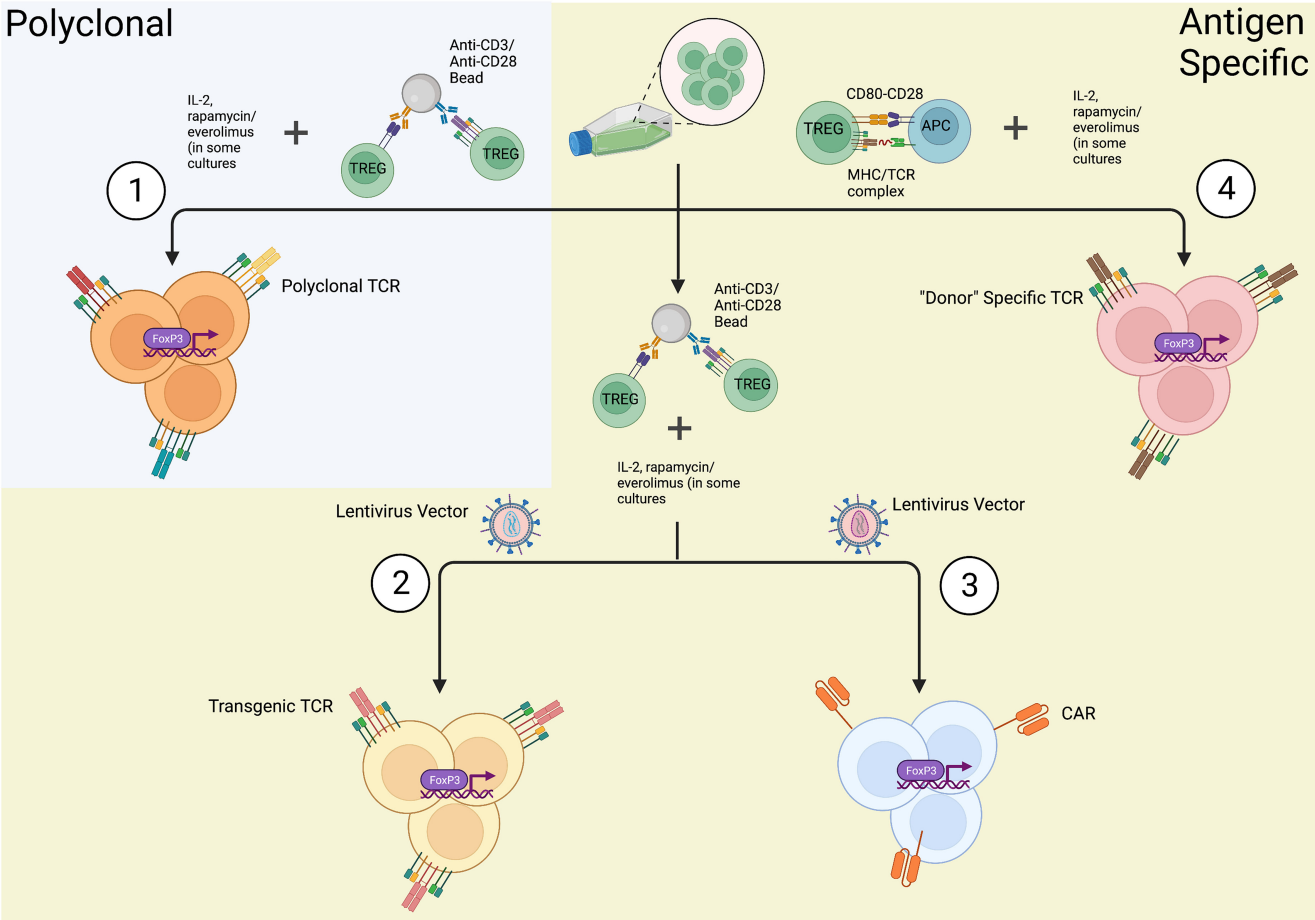
*In-vitro* methods of Treg expansions. Once Tregs are isolated from a source utilizing FACS or immunomagnetic sorting, they can be expanded in a (1) polyclonal fashion or (2-4) antigen specific manner. (1) Tregs are polyclonally expanded in the presence of anti-CD3/anti-CD28 antibodies resulting in mature Tregs with the expression of a diverse array of T-cell receptors (TCRs). (2) Genetically engineered Tregs undergo polyclonal expansion and also transfection with a lentiviral/retroviral vector coding for a specific TCR producing Tregs expressing only one specific TCR. (3) Chimeric antigen receptor (CAR) Tregs are expanded *via* polyclonal expansion and transfection with lentiviral/retroviral vectors expressing CARs fused to intracellular signaling domains. (4) In the context of transplantation, “Donor” specific Tregs can be expanded *via* co-culture with activated, allospecific antigen presenting cells (APCs). As depicted, these *in-vitro* methods of Treg expansion additionally require supplementation of culture media with IL-2 and other Treg inducing molecules, such as rapamycin or everolimus, in some culture protocols (created with Biorender.com).

Following isolation, Tregs are expanded *in-vitro* to reach numbers suitable for clinical use (**Figure 3**). A reliable method of expansion was first described by Hoffman et al. in 2004 (130). The group successfully expanded polyclonal Tregs up to 40,000 fold using magnetic beads with attached antibodies specific for CD3 and CD28 and demonstrated Tregs retained suppressive capacity as measured through mixed lymphocyte reactions (MLR). This is still the most commonly used method today and is capable of producing large numbers of polyclonal Tregs. A downside of this technique though is that the beads must be removed prior to clinical use, which may affect both their total number and purity. To combat this problem, companies have developed soluble reagents such as the T cell TransAct (Miltenyi Biotec), which is a polymer nanomatrix conjugated with antibodies to CD3/CD28, or soluble tetrameric antibody complexes that bind CD3, CD28 and CD2 (Stemcell Technologies). Artificially engineered APCs expressing CD64 or CD86 and anti-CD3 antibody have been used to expand Tregs *in vitro* followed by irradiation to remove them prior to their use (131, 132). IL-2 is used in expansion protocols due to its role in activating Tregs through the CD25 receptor, and some groups have supplemented rapamycin to the expansion media (123–135) given its ability to suppress effector T cell proliferation and increase the suppressive ability of Tregs (123, 136). Specifically, following isolation of Tregs *via* the methods described above, cells were cultured in medium supplemented with human serum, IL-2 and 100nm of rapamycin. Lombardi et al. performed a series of stimulations with anti-CD3/CD28 activation beads every 12 days with final cell harvesting on day 36 of culture. Rapamycin and IL-2 were replenished every 2-3 days throughout the culture. At the end of a 36 day culture, Tregs expanded over 6,000 fold in the presence of rapamycin with greater suppressive capability (123). Battaglia et al. cultured isolated Tregs in a similar fashion with 100nM rapamycin, but performed a series of 3 stimulations every 7 days with a final harvest on day 28 of culture. Expanded Tregs exhibited suppressive ability and sustained Foxp3 expression (135). More recently, everolimus, a rapamycin derivative, has been shown to yield a more pure Treg product (136).

Despite the convenience and advances in the production of polyclonally expanded Tregs, it is important to note that polyclonally expanded Tregs are known to cross react with a broad repertoire of non-specific targets. Furthermore, the regulatory function and stability of polyclonal Tregs produced in large scales to fit the clinical setting still remains a challenge. This led to the development of antigen specific Tregs for a more targeted approach of immune tolerance. Putnam et al. was one of the first groups to develop a protocol to isolate and expand antigen specific Tregs in humans (113). The group generated virally transfected K562 B cells expressing CD40 ligand (CD40L) which were then used to stimulate isolated Tregs. Tregs were then restimulated with a polyclonal expansion (CD3/CD28 beads). Results demonstrated a 100- to 1600-fold expansion of antigen specific Tregs with highly potent suppressive activity both *in vitro* and in a humanized mouse model. Todo et al. successfully developed a simple and inexpensive *ex-vivo* method to expand antigen specific Tregs by coculturing recipient lymphocytes with irradiated donor cells in the presence of anti-CD80/CD86 monoclonal antibodies (137). More recently, Mathew et al. established a protocol to culture and expand antigen specific Tregs at a large scale (126). Donor APCs (B cells) were stimulated and cultured using a cell free, soluble CD40L tetramer (Ultra-CD40L) followed by stimulation of recipient Tregs with the cultured donor B cells. The group achieved a 100-fold expansion of antigen specific Tregs with strong inhibition activity against recipient T cells. MLR assays with a Treg to responder cell ratio of (1:10) to (1:250), demonstrated the presence of significant recipient cell growth inhibition induced by antigen specific Tregs (126). The benefit of using antigen specific Tregs as compared to polyclonally expanded Tregs is in their ability to induce highly specific immunosuppression with fewer numbers of cells; however, assumptions about the absence of off-targeted immunosuppression by antigen specific Tregs cannot be assumed (138). Additionally, Treg expansion using donor APCs results in low yield of post expansion cells due to the limited antigen specific Tregs present within the entire Treg population of an individual. Hence, genetically modified antigen specific Tregs using technologies, such as a trans-genetically expressed T-cell receptors (TCRs) and chimeric antigen receptors (CARs) are being developed.

**Figure 4:**
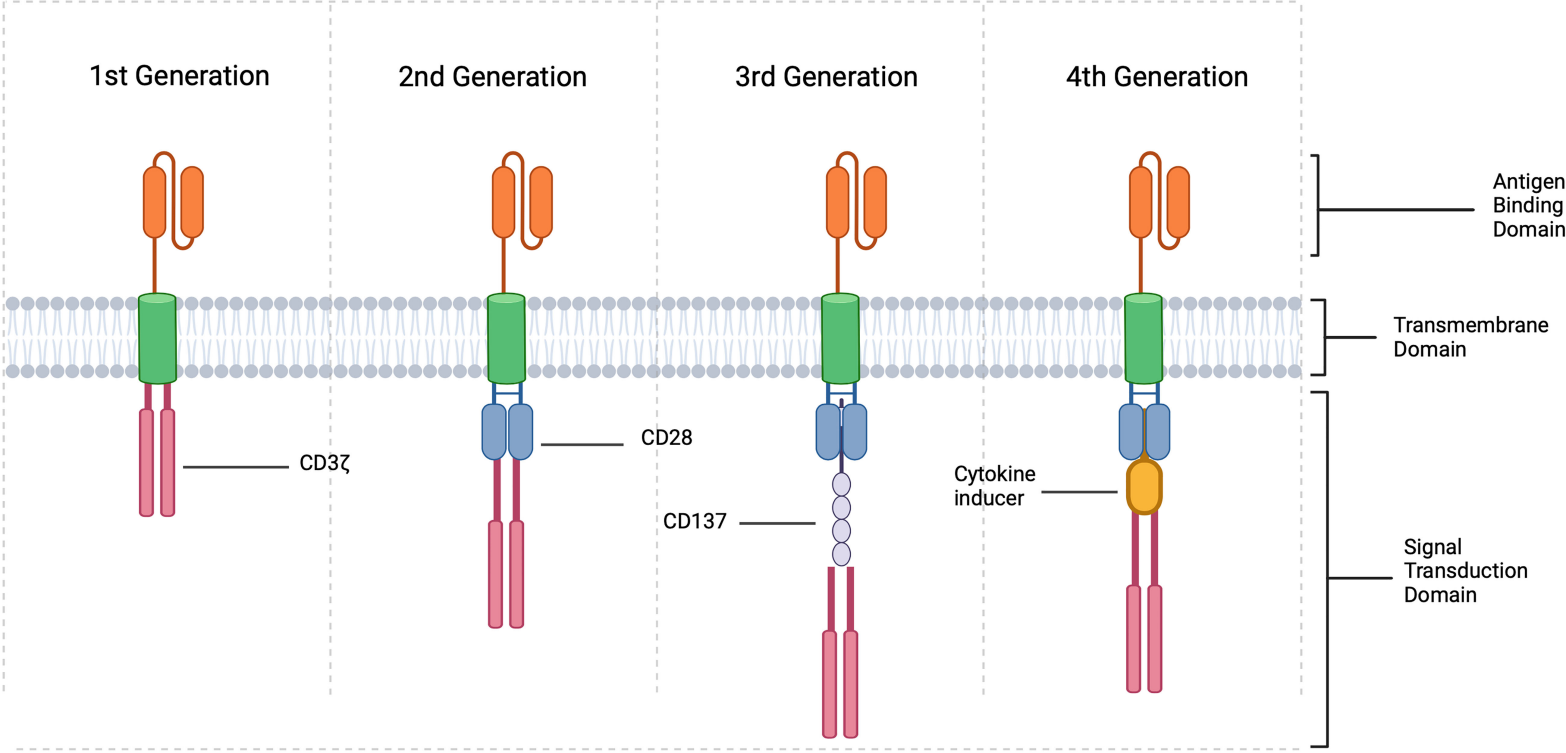
CAR Treg constructions. CARs are created by fusing a specific antigen binding domain to a transmembrane domain and a single or multiple intracellular signaling domains responsible for T cell activation. In the context of Tregs, 1^st^ generation include an intracellular CD3 ζ signaling domain, 2^nd^ generation consist of intracellular CD3 ζ and typically CD28 and 3^rd^ generation CD3 ζ, CD28 and CD137 or other co-stimulatory molecule. 4^th^ generation CARs are created by modifying 2^nd^ or 3^rd^ generation CARs with the addition of a cytokine inducing domain, suicide gene, or safety switch (created with Biorender.com).

In order to accomplish this, TCR genes must first be identified *via* whole transcriptome sequencing. Once a disease specific TCR gene is determined, it can be introduced into the cell line of choice either through retroviral or lentiviral vector transduction. This was first accomplished in humans in an attempt to develop cytotoxic T cells in the treatment of melanoma (139), and the technology is now being utilized to engineer Tregs for treatment of other disease states, such as autoimmunity. Though, this technology is limited based on MHC class recognition and may not be very effective in certain scenarios. This limitation led to the use of the CAR cell, which is one of the most innovative breakthroughs in the field of cellular therapeutics. As demonstrated in **Figure 4**, CARs are receptors synthesized from the fusion of artificial proteins comprising of a transmembrane domain linked to an extracellular antigen recognition domain (e.g., single-chain variable fragments, scFv) and to an intracellular signal transduction domain(s) (domains responsible for the activation of T cells) (140–142). The intracellular signal transduction domain consists of a main signaling chain (e.g., TCR CD3ζ signaling chain) and co-stimulatory chains (e.g., CD28 signaling molecules) (140). To date, three generations of CARs-Tregs have been synthesized. CARs were initially generated with a single signaling chain, namely TCR CD3ζ. The 2^nd^ generation of CARs contained co-stimulatory chains such as CD28 signaling molecules, and 3^rd^ generation CARs were further modified with additional co-stimulatory chains (140). 4^th^ generation CARs are now being created through the modification of a 2^nd^ or 3^rd^ generation CAR molecule to include a specific cytokine inducing transgene, suicide gene or safety switch to improve the function and/or safety of the cellular therapy. For example, including an inducer of IL-10 or TGF- β could potentially improve the efficacy and suppression exhibited by CAR-Tregs while also modulating the inflammatory environment within an allograft. These constructs are then used to create lentiviral or retroviral vectors that are transduced into the cell line of choice for final expression. The extracellular domain can recognize antigens expressed on the surface of donor APCs or additionally recognize soluble antigens, leading to the activation of the cell. Significant advances have been made in the development of adoptive cell transfer techniques, and these technologies are now being extensively studied in the treatment of human disease.

## *In vivo* expansion and Foxp3+ Treg-based therapies in autoimmunity

A great deal of research has gone into Treg therapies in the context of autoimmune disease with the hopes of restoring immune tolerance without the broad immunosuppressive effects observed with current treatment modalities. IL-2 is an important signaling molecule and is recognized by CD25 expressed on the surface of Tregs. Low dose IL-2 has been shown to preferentially expand CD25 expressing Tregs in autoimmune disease (143). Additionally, its safety and efficacy as a therapy has been shown in both SLE and type 1 diabetes (144–148). Further modifications of IL-2 have been pursued including use of IL-2 for the *ex-vivo* expansion of Tregs and also in the development of Tregs that express a modified IL-2 receptor that recognizes an engineered IL-2 molecule (IL-2 muteins) (149). Rapamycin, a potent mammalian target of rapamycin (mTor) inhibitor, has been shown to block T cell cycle progression and selectively expand Tregs (150). Battaglia et al. showed that tolerance could be achieved *via* co-administration of both IL-2 and rapamycin in a diabetic mouse model (151). This combination was also shown to suppress effector T cell activation *via* restoration and maintenance of the Th17/Treg balance in refractory SLE patients, which was associated with less disease activity as measured by the systemic lupus erythematosus disease activity index (SLEDAI) (152). Administration of autoantigens through vaccination, oral, intra-nasal, subcutaneous and intravenous injections have been studied (153, 154). Serra et al. took this a step further and developed a nanoparticle technology to deliver autoantigens in order to promote formation of tolerogenic dendritic cells and expansion of Tregs *in vivo* (155). Methotrexate loaded nanoparticles were shown to decrease levels of tumor necrosis factor alpha, vascular endothelial growth factor and IL-1β in an experimental mouse model for rheumatoid arthritis. Increased numbers of Tregs were identified in mice treated with loaded nanoparticles compared to those mice that received methotrexate alone (156). These therapies have had good results, but more promising are the adoptive cell based therapies utilizing *ex-vivo* expanded Tregs and CAR-Tregs as discussed above.

Mottet et al. was one of the first groups to show that transfer of CD4+CD25+ Tregs could successfully treat colitis in immune deficient mice within two weeks of infusion (157). Using a mouse CD4+CD45RB^High^ T cell transfer model for colitis, the group demonstrated that the transfer of CD4+CD25+ T cells ameliorated both clinical and histological signs of colitis after a single infusion. In contrast, mice that received either CD4+CD25- T cells or no transferred T cells showed no clinical improvement and had persistent signs of inflammation on colonic histology (157). In a follow-up study, CD4+CD25+Foxp3+ cells were found to be present in similar frequencies within draining lymph nodes and the lamina propria of colitic mice suggesting both localized and systemic regulatory effects. They further determined that cure of colitis was IL-10 dependent, and that IL-10 producing CD4+CD25+ Tregs were mainly located in the lamina propria of the intestine suggesting functional compartmentalization of CD4+CD25+ T cells during disease states (158). Analysis of human IBD specimens found similar results with an accumulation of CD4+CD25+Foxp3+ within the inflamed intestine of patients. Transfer of CD4+CD25+ T cells has also been shown to cure colitis in a *SCID* mouse CD4+CD25- T cell transfer model with concomitant infection with *Leishmania major* (159). Improvement in colitis was found to be IL-10, TGF-β, and CTLA-4 dependent after administration of antagonistic IL-10 receptor, TGF-β, and CTLA-4 antibodies (159). In 2014, one of the first clinical trials utilizing *ex vivo* expanded polyclonal Tregs in children with type 1 diabetes mellitus was completed. In this study, administration of polyclonal Tregs resulted in prolonged survival of pancreatic islets with signs of clinical remission and insulin independence up to 1 year post-infusion (129, 160). Since that time, multiple other clinical trials investigating the use of Tregs in autoimmune disease have been completed or are currently active (**Table 1**). Although these studies showed that polyclonal Tregs are safe and effective, there still exists a theoretical risk of inadvertent widespread immunosuppression given their lack of antigen specificity. As such, the use of antigen specific Tregs has garnered more interest. These antigen specific Tregs have greater ability to achieve tolerance compared to their polyclonal counterparts, and are capable of resulting in complete remission of autoimmune disease. Tang et al. showed that in a mouse model of type 1 diabetes mellitus a lower number of antigen specific Tregs were needed to prevent and in some cases reverse the disease process (170). Desreumaux et al. was the first group to adoptively transfer antigen-specific Tregs in the management of Crohn’s disease (165). In a 12-week, randomized trial of 20 patients with refractory Crohn’s Disease, an infusion of ovalbumin-specific Tregs resulted in a dose related improvement in disease severity as measured by the Crohn’s Disease Activity Index (CDAI).

**Table 1:** Active or completed Treg clinical trials in both autoimmune disease and solid organ transplantation as published on clinicaltrials.gov.

Autoimmune Disease				
NCT Number	Acronym	Phase	Disease	Cell Type
NCT05349591	cePolyTregs	Phase 1	Type 1 Diabetes	Autologous, polyclonal
NCT04820270 (161)		Phase 1	Type 1 Diabetes	Autologous, polyclonal
NCT04691232 (162)		Phase 1	Ulcerative Colitis	Autologous, polyclonal
NCT03444064		Phase 1	Type 1 Diabetes	Autologous, polyclonal
NCT03239470	PolyTregs	Phase 1	Pemphigus	Autologous, polyclonal
NCT03162237		Phase 1	Type 1 Diabetes	Autologous, polyclonal
NCT03011021		Phase 1|Phase 2	Type 1 Diabetes	Autologous, polyclonal
NCT02932826		Phase 1|Phase 2	Type 1 Diabetes	Autologous, polyclonal
NCT02772679 (163)	TILT	Phase 1	Type 1 Diabetes	Autologous, polyclonal
NCT01210664 (164)	Treg	Phase 1	Type 1 Diabetes	Autologous, polyclonal
Eudract 2006-004712-44 (165)		Phase 1|Phase 2	Crohn’s Disease	Autologous, antigen-specific

Transplantation				
NCT Number	Acronym	Phase	Disease	Cell Type
NCT02088931 (128)	TASKp	Phase 1	Kidney Transplant	autologous, polyclonal
NCT02091232 (166–168)	ONE Study	Phase 1	Kidney Transplant	autologous, antigen specific
NCT02474199	ARTEMIS	Phase 1|Phase 2	Liver Transplant	autologous, antigen specific
NCT02145325 (125)	TRACT	Phase 1	Kidney Transplant	autologous, polyclonal
NCT02166177	ThRIL	Phase 1|Phase 2	Kidney Transplant	autologous, polyclonal
NCT02371434 (166, 168)	ONEnTreg13	Phase 1|Phase 2	Kidney Transplant	autologous, polyclonal
NCT02244801 (166)	DART	Phase 1	Kidney Transplant	autologous, antigen specific
NCT02129881 (166, 167)	ONETreg1	Phase 1|Phase 2	Kidney Transplant	autologous, polyclonal
NCT03654040	LITTMUS-UCSF	Phase 1|Phase 2	Liver Transplant	autologous, antigen specific
NCT03577431	LITTMUS-MGH	Phase 1|Phase 2	Liver Transplant	autologous, antigen specific
NCT04924491 (169)	THYTECH	Phase 1|Phase 2	Heart Transplant	autologous, thymic
NCT03943238		Phase 1	Kidney Transplant	autologous polyclonal
NCT04817774	STeadfast	Phase 1|Phase 2	Kidney Transplant	autologous, CAR/antigen specific
NCT02711826	TASK	Phase 1|Phase 2	Kidney Transplant	autologous, polyclonal
NCT05234190	LIBERATE	Phase 1|Phase 2	Liver Transplant	autologous, CAR/antigen specific
NCT03867617		Phase 1|Phase 2	Kidney Transplant	autologous, polyclonal
NCT03444064		Phase 1	Diabetes	autologous, polyclonal
NCT05349591	cePolyTregs	Phase 1	Diabetes	autologous, polyclonal
NCT03284242		Phase 1	Kidney Transplant	autologous, polyclonal
NCT04820270 (161)		Phase 1	Diabetes	autologous, polyclonal

Trials listed below without an associated reference have no published results to date.

In some disease states, levels of naturally occurring antigen-specific Tregs are low which can present a challenge during *ex-vivo* expansion. In an effort to avoid this problem, genetically engineered Tregs expressing a specific TCR have been developed and used to target type 1 diabetes mellitus, multiple sclerosis, and acquired factor VIII Deficiency *in vitro* (171–173). Another technology that circumvents the potentially low number of circulating disease specific Tregs is the CAR Treg. CAR Tregs possess the ability to be both antigen specific and migrate to the area of disease where their suppressive functions can be focused (174). In colitis, Elinav et al. was the first to prevent 2,4,6-trinitrobenzene sulfonic acid (TNBS) induced colitis in transgenic mice expressing T cells with a TNP-CAR molecule (175). They later confirmed these findings through the adoptive transfer of only CAR-TNP Tregs which prevented colitis and improved survival (175). CAR Tregs expressing carcinoembryonic antigen have been studied in mouse models, and have been found to localize to the colon, prevent colitis, and also suppress development of colitis associated cancer (176). In type 1 DM, Imam et al. developed CAR constructs against 2 epitopes of GAD 65 which were then utilized in *ex-vivo* expanded Tregs. In a humanized type 1 diabetic mouse model, CAR-Tregs infiltrated pancreatic islets within 24 hours of infusion and those mice that received CAR-Tregs showed lower blood glucose by post-infusion day 30 (177). Despite these encouraging results, a potential downside of this technology is that the antigen of interest must only be expressed at the site of disease, as there is a risk of broad immune suppression if the antigen is expressed throughout the body (178). In the setting of autoimmune disease, this poses a particular challenge given the lack of currently known specific antigens for each disease state. As such, further investigation is required before these modalities are implemented for widespread use in humans.

## Foxp3+ Treg-based therapies in solid organ transplantation

Since it was demonstrated that CD4+CD25+T cells could prevent the onset of autoimmune disease in thymectomized mice (13), they have been extensively studied as a treatment modality following solid organ transplantation. The efficiency of Tregs in inducing immune tolerance among transplant patients is affected by the choice and duration of IS regimens administered following transplantation (179–181). For example, an increase in CD4+CD25+Foxp3+ peripheral Treg populations was observed in liver transplant recipients following administration of mTor inhibitors when compared to tacrolimus and other calcineurin inhibitors (2, 179). Further, the presence of pro-proliferative activity within Tregs in the periphery demonstrated preservation of their viability and quality (2, 180). Pro-inflammatory proteins responsible for lymphocyte activation, adhesion and dendritic cell activation were downregulated whereas anti-inflammatory proteins responsible for the protection of kidney grafts were upregulated when mTor inhibitors were used (2). Tacrolimus in combination with Mycophenolate mofetil (MMF) was shown to increase expression of Tregs and other Treg enhancing cytokines such as IL-10 and TGF-β when compared to Tacrolimus alone in liver transplant patients (181). Given the substantial effect IS regimens can have on Treg numbers and efficacy, the development of an optimized protocol to promote Treg activity is needed. However, adoptive cellular therapies such as those described above are currently being optimized for the use in solid organ transplantation and may be an enticing solution.

In 2020, Sánchez‐Fueyo et al. conducted a Phase 1 clinical trial in adult deceased donor liver transplant patients who intravenously received a single dose of autologous, polyclonally expanded Tregs, 3 to 16 months post transplantation. Tregs were isolated from whole blood or leukapheresis products and were successfully expanded *via* Good Manufacturing Practice (GMP) protocols (182). Excluding one patient who developed a dose-dependent toxic reaction, there were no observed infectious complications (182). The absence of anti-donor T cell responses post-transplant confirmed the pro-immunosuppressive capability of the infused Tregs (182). Similarly, Mathew et al. demonstrated the safety of autologous, polyclonally expanded Tregs (125). In a Phase 1 dose escalation trial (TRACT), kidney transplant recipients were administered a single infusion of autologous, polyclonally expanded Tregs on post-operative day 60 following the conversion of immunosuppression from tacrolimus to sirolimus. There were no adverse events observed up to two years post-transplant. Further, there was a tremendous and sustained increase in the percentage and absolute number of circulating Tregs after the infusion. This may have been mediated by infectious tolerance as the expanded Tregs were found to generate new Tregs in autologous responding cells (125). The TASK Trial investigated the safety of polyclonal Tregs in patients with subclinical graft inflammation at 6 months post-kidney transplant (128). The group demonstrated no significant adverse events related to therapy and also showed comparable persistence and stability of Treg populations in subjects receiving standard immunosuppression versus those who were not receiving immunosuppression (128). The ONE study was the combined effort of seven, single armed trials studying the safety and efficacy of adoptive cell therapy in living donor kidney transplant recipients including polyclonal Tregs and antigen specific Tregs, as well as dendritic cell and macrophage cell products (166–168). The combined data showed comparable 60-week rejection rates between the standard immunosuppression group (12%) and six cell-based therapy groups (16%) with 40% of those receiving cellular therapy successfully weaned off of MMF and maintained only on tacrolimus (166). There were no increased rates of adverse events and lower reported infectious rates in the cell-based therapy groups (166).

Antigen specific Tregs have been shown to suppress the proliferation of alloreactive T cells and prolong the survival of mice allografts as compared to non-antigen specific Tregs (183). The higher inhibition against donor cells as compared to non-specific third-party cells, highlights the superiority of antigen specific Tregs’ application in solid organ transplants as compared to polyclonally expanded Tregs (137, 183). As described above, Todo et al. successfully developed an *ex-vivo* method to expand donor specific Tregs by co-culturing recipient lymphocytes with irradiated donor cells in the presence of anti-CD80/CD86 monoclonal antibodies (137). These donor specific Tregs were then studied in one of the first clinical trials using adoptively transferred Tregs in liver transplant recipients. Thirteen days following liver transplantation, recipients were administered a single infusion of donor specific Tregs. Patients additionally underwent splenectomy at the time of transplant and were weaned off of immunosuppression starting at 6 months post-transplant (MMF, tacrolimus). Overall, 70% of recipients were completely weaned from immunosuppressants for 16 – 33 months, and the dosage of immunosuppressants was reduced in 30% of recipients due to the presence of mild rejection following immunosuppressant withdrawal (137). It must be mentioned that the three patients who experienced rejection were transplanted for autoimmune hepatitis, suggesting the threshold to achieve tolerance may be higher in those individuals with an underlying defect leading to loss of self-tolerance. Multiple other clinical trials utilizing both polyclonally expanded and antigen specific Tregs are currently in progress or have been completed and are summarized in **Table 1**.

The targeted cytotoxicity and growth inhibition effects of CAR-T cells against specific cancers has led to the utilization of CAR-Tregs in the field of solid organ transplantation. As CAR-conjugated cells are able to offer a targeted approach, the creation of CAR-Tregs could encourage accelerated homing of antigen specific Tregs to a transplanted graft (141, 184). CAR-Tregs have been shown to inhibit the proliferation of alloreactive cells and prevent graft rejection to a greater extent when compared to polyclonal Tregs both *in vitro* and *in vivo* xenograft transplant models, respectively. In the context of transplantation, CAR-Tregs are engineered with a focus on the HLA-A2 allele, as this allele family is positive in approximately 50% of the population (184, 185). Therefore, the benefits of HLA-A2 CAR-Tregs in preventing graft rejection could be reaped by HLA-A2 positive donors and HLA-A2 negative recipients in the transplant setting (185). HLA-A2 CAR-Tregs synthesized using scFv regions from BB7.2 mice monoclonal antibodies linked to Treg associated co-stimulatory molecules (CD8, CD28 and CD3ζ) exhibited significant antigen specific anti-proliferative effects against alloreactive CD8+ T cells (186). In another study, HLA-A2 CAR-Tregs developed using a human-derived HLA-A2 specific scFv sequence linked to co-stimulatory molecules (CD28 and CD3ζ) showed greater suppression of effector T cells when compared to polyclonal Tregs (184). Accelerated migration, increased infiltration of CAR-Tregs in a targeted manner and reduction of tissue damage was also observed in an HLA-A2 specific skin allograft model using these same CAR-Tregs (184). This phenomenon highlights the benefit of targeted immunosuppression provided by antigen specific CAR-Tregs. CAR-Tregs have been shown to increase the production of immunosuppressive cytokines such as IL-10 and prevent targeting of anti-inflammatory cells for cell death. These findings suggest that CAR Tregs could create an anti-inflammatory milieu in both the graft and surrounding matrix (184). Phenotypically and functionally stable fluorescein isothiocyanate (FITC) conjugated monoclonal antibody CAR-Tregs suppressed the proliferation of alloreactive lymphocytes when studied *in vitro* and minimized the infiltration of alloreactive CD8+ lymphocytes and prolonged the survival of islet cells in an allogenic murine islet transplant model (187). The group went further and showed prolonged survival of secondarily transplanted skin allografts through the activity of infused CAR-Tregs. More recently, Schreeb et al. established a phase I/IIa study protocol (STEADFAST) to expand and infuse recipient HLA-A2 CAR-CD4^+^/CD45RA^+^/CD25^+^/CD127^low/neg^ Tregs into HLA-A2 negative end-stage renal disease recipients (188). Consistent with previous findings, infused HLA-A2 CAR-Tregs are anticipated to migrate to and bind to the specifically targeted HLA-A2 positive allograft. The study is currently in progress and no results have been published at this time.

## Challenges and shortcomings of adoptive cell therapy

Despite the promising results of the above studies, further research is required to reliably utilize Treg adoptive cell therapies in autoimmune disease and solid organ transplantation. Polyclonal Tregs, while easy to produce in large numbers *in vitro*, have the potential to promote widespread immunosuppression given their lack of antigen specificity. On the contrary, antigen specific Tregs are more effective in inhibiting alloimmune response compared to polyclonal Tregs, but the effort to culture and expand antigen specific Tregs in a large-scale clinical setting still remains a challenge. This is largely a result of the paucity of disease specific Tregs present in peripheral blood, which may lead to long and costly *ex vivo* expansions with a lower quality Treg product. Further, there are numerous autoimmune diseases that we have still not identified a disease specific antigen to be targeted. Although Tregs engineered using CAR technology have been proven to be the most efficient approach in terms of maintaining immune homeostasis among transplant recipients due to greater phenotypic stability (184, 186), the long-term viability, stability and functionality of all types of *ex-vivo* expanded Tregs post infusion is an area of concern. More specifically, Tregs are known to have plasticity in inflammatory environments with the potential to convert into an effector T cell phenotype. As such, it is imperative to investigate other means of stabilizing infused Tregs *in vivo.* Such approaches could include forced over-expression of Foxp3, silencing of the IL-17 gene, administration of immunomodulatory agents such as anti-CD3 molecules, or use of IL-2 muteins as discussed above. Specific to CAR Tregs, some have advocated for conjugation of particles to CAR-Tregs in an effort to induce release of Treg pro-proliferating growth cytokines with homing antibodies specific to the target allograft (189, 190), and conversely building in suicide genes in the event CAR-Tregs malfunction and become neoplastic (191). Another difficulty is that certain disease states may have multiple disease specific antigens, affecting not only the overall suppressive activity of Tregs but also their ability to migrate to tissues of interest. One potential solution is to engineer Tregs that express homing receptor markers for certain disease states, an area of research that is still left largely unexplored. Finally, in certain diseases, the Tregs may have inherent defects that could not be rectified with *ex-vivo* expansions, resulting in ineffective Treg products. Therefore, there is a great need to further investigate Treg subsets in specific diseases and develop personalized Treg products to ensure the highest quality Treg for adoptive transfer.

## Conclusion

Regulatory T cells are critical for the maintenance of tolerance in humans. The mechanisms by which they contribute to immune homeostasis are complex, and malfunction of even one of these pathways can lead to inadequate regulatory activity and the development of disease. In autoimmune disease, this may be a result of germline genetic mutations or disease specific inflammatory cytokines at local sites of inflammation, and in the setting of solid organ transplantation this is secondary to donor antigens presented *via* the direct, semi-direct, or indirect allorecognition pathways. For both, adoptive Treg cell transfer is an exciting technology that could revolutionize the care of these patients. Although much progress has been made, additional studies must be undertaken with a focus on further characterizing the safety and efficacy of Treg adoptive cell technology in human diseases, identifying disease specific antigens and Treg subsets, and developing strategies to improve the *in vivo* stability and suppressive function of adoptively transferred Tregs to ensure a long-lasting effect with few adverse reactions.

## Author contributions

All authors contributed to the article and approved the submitted version.

## Funding

The authors were supported by the Frankel Family Foundation. Research reported in this publication was also supported by the National Institute of Diabetes and Digestive and Kidney Diseases of the National Institutes of Health under Award Number T32DK077662 (Grant: T32DK077662/TR/NIAID NIH HHS/United States). The content is solely the responsibility of the authors and does not necessarily represent the official views of the National Institutes of Health.

## Acknowledgments

All figures in this manuscript were created using biorender.com.

## Conflict of interest

The authors declare that the research was conducted in the absence of any commercial or financial relationships that could be construed as a potential conflict of interest.

## Publisher’s note

All claims expressed in this article are solely those of the authors and do not necessarily represent those of their affiliated organizations, or those of the publisher, the editors and the reviewers. Any product that may be evaluated in this article, or claim that may be made by its manufacturer, is not guaranteed or endorsed by the publisher.

## Abbreviations

SOT, solid organ transplantation; IS, immunosuppressive; SLE, systemic lupus erythematosus; Tregs, regulatory T cells; Foxp3, forkhead box p3; tTregs, thymic derived regulatory T cells; pTregs, peripherally derived regulatory T cells; APC(s), antigen presenting cells; TGF- β, transforming growth factor beta; MHC, major histocompatibility complex; FACS, fluorescence activated cell sorting; MLR, mixed lymphocyte reactions; TCR(s), T cell receptors; CAR(s), chimeric antigen receptors; mTOR, mammalian target of rapamycin; TNBS, 2,4,6-trinitrobenzene sulfonic acid; MMF, mycophenolate mofetil; GMP, good manufacturing practice.
